# Circular RNA hsa_circ_0061395 accelerates hepatocellular carcinoma progression via regulation of the miR-877-5p/PIK3R3 axis

**DOI:** 10.1186/s12935-020-01695-w

**Published:** 2021-01-06

**Authors:** Yanhui Yu, Lijuan Bian, Renfei Liu, Yitong Wang, Xia Xiao

**Affiliations:** 1 Department of Clinical Laboratory, The Second Hospital of Jilin University, No. 218ZiQiang Street, Changchun, 130001 Jilin China; 2 Department of Cataract, Eye Center, The Second Hospital of Jilin University, Changchun, Jilin China; 3 Department of Health, Medical Reform Management Office, Changchun, Jilin China

## Abstract

**Background:**

Circular RNA hsa_circ_0061395 (circ_0061395) has been reported to accelerate the advancement of hepatocellular carcinoma (HCC). However, the regulatory mechanism by which circ_0061395 modulates the progression of HCC is unclear.

**Methods:**

The morphology and size of exosomes were analyzed by transmission electron microscope (TEM) and nanoparticle-tracking analysis (NTA). Protein levels were detected by western blotting. Expression levels of circ_0061395, microRNA (miR)-877-5p, and phosphoinositide-3-kinase regulatory subunit 3 (PIK3R3) mRNA were assessed by quantitative real time polymerase chain reaction (qRT-PCR). The proliferation, invasion, migration, cell cycle progression, and apoptosis were analyzed by cell counting kit-8 (CCK-8), plate clone, transwell, or flow cytometry assays. The targeting relationship between circ_0061395 or PIK3R3 and miR-877-5p was verified using the dual-luciferase reporter and/or RNA immunoprecipitation (RIP) assays. Xenograft assay was performed to confirm the biological function of circ_0061395 in HCC.

**Results:**

Circ_0061395 was upregulated in HCC tissues, serum, cells, and serum-derived exosomes. Circ_0061395 silencing decreased tumor growth in vivo, and induced cell cycle arrest, apoptosis, repressed proliferation, invasion, and migration of HCC cells in vitro. MiR-877-5p was downregulated while PIK3R3 was upregulated in HCC. Circ_0061395 regulated PIK3R3 expression via competitively binding to miR-877-5p. MiR-877-5p inhibitor overturned circ_0061395 knockdown-mediated influence on malignant behaviors of HCC cells. PIK3R3 overexpression reversed the suppressive influence of miR-877-5p mimic on malignant behaviors of HCC cells.

**Conclusion:**

Circ_0061395 facilitated HCC progression via regulating the miR-877-5p/PIK3R3 axis, providing a new perspective on the advancement of HCC.

## Highlights

1. Circ_0061395 was highly expressed in HCC tissues, serum, cells, and serum-derived exosomes.
2. Inhibition of circ_0061395 reduced the malignancy of HCC cells.
3. Circ_0061395 acted as a sponge for miR-877-5p in HCC cells.
4. PIK3R3 served as a target for miR-877-5p in HCC cells.
5. Circ_0061395 sponged miR-877-5p to regulate PIK3R3 expression.

## Introduction

Liver cancer ranks fourth among cancer-related death causes globally [1]. Hepatocellular carcinoma (HCC) is one of the most common pathological types of primary liver cancer, accounting for about 85% of all liver cancer cases [2]. Although new breakthroughs have been made in the treatment of HCC, the 5-year survival rate is still low [3, 4]. Hence, studying the underlying molecular mechanisms of HCC advancement can provide potential strategies for the treatment of HCC.

Circular RNAs (CircRNAs) are a type of endogenous RNA molecules with the characteristics of covalently closed loops [5]. Unlike liner transcripts, circRNAs are more resistant to RNase R due to their lack of 5′ cap and 3′ poly(A) tail [6]. Some circRNAs can act as competing endogenous RNAs (ceRNAs) and regulate gene expression by interacting with microRNAs (miRs) [7]. Moreover, circRNAs have been revealed to be implicated in the advancement of diverse tumors [8]. For instance, pancreatic cancer cells-secreted exosomal circRNA_IARS accelerated tumor metastasis via regulation of the permeability of the endothelial cell monolayer [9]. CircRNA hsa_circ_0061395 (circ_0061395), located at chromosome 21: 30698379–3070201, is formed by head-to-tail splicing of exons 3 and 4 of the BTB domain and CNC homolog 1 (BACH1) gene (http://www.circbank.cn/search.html?selectValue=hsa_circ_0061395). It was reported that circ_0061395 facilitated HCC progression via inhibiting p27 expression [10]. However, the molecular mechanism that circ_0061395 promotes HCC progression has not been fully explained.

MiRs control protein production via partly complementary binding of the mRNA 3′ untranslated regions (UTRs) [11]. Studies have demonstrated that miRs exert vital regulatory roles in cellular processes, such as cell differentiation, invasion, migration, autophagy, and apoptosis [12]. MiR-877-5p had been proved to play an anti-tumor impact on cervical cancer [13] and glioblastoma [14]. Also, miR-877-5p constrained malignant behaviors of HCC cells [15]. Nevertheless, the specific mechanism by which miR-877-5p regulates HCC advancement is still unclear.

Phosphoinositide-3-kinase regulatory subunit 3 (PIK3R3), a regulatory subunit of phosphoinositide-3-kinase, participates in multiple signal pathways [16]. PIK3R3 had been reported to be upregulated in diverse tumors, such as ovarian cancer [17], colorectal cancer [18], and glioma [19]. Furthermore, PIK3R3 has proven to be an oncogene in HCC [20, 21]. However, whether PIK3R3 can be regulated by circ_0061395 and miR-877-5p in HCC progression is indistinct.

In the present study, circ_0061395 was upregulated in HCC tissues, serum, cells, and serum-derived exosomes. Mechanically, circ_0061395 facilitated HCC progression via elevating PIK3R3 expression via competitively binding to miR-877-5p, which provided a new insight into for the advancement of HCC.

## Materials and methods

### Human blood and tissue samples

Human blood and/or tissue samples (HCC tissues and adjacent non-tumor tissues) were collected from 30 HCC patients and 30 healthy controls that were administrated at the Second Hospital of Jilin University. These blood samples were centrifuged at 1200×*g* for 20 min to obtain serum samples. The research was approved by the Ethics Committee of the Second Hospital of Jilin University. All recruited participants (HCC patients and healthy controls) signed written informed consents.

### Cell culture

293T cells, normal adult liver epithelial cells (THLE-2), and HCC cell lines (SNU-387 and Huh-7) were purchased from BeNa Culture Collection (Suzhou, Jiangsu, China). These cells were cultured in a moist atmosphere with 5% CO_2_ at 37˚C. THLE-2 cells were cultured using bronchial epithelial cell growth media (Clonetics Corporation, Walkersville, MD, USA). 293T, Huh-7, and SNU-387 cell lines were cultured in Dulbecco’s modified Eagle’s medium (DMEM) (Sigma, St Louis, MO, USA) (for 293T and Huh-7 cells) or Roswell Park Memorial Institute (RPMI)-1640 medium (Sigma) (for SNU-387 cells) supplemented with fetal bovine serum (FBS, 10%, Solarbio, Beijing, China) and streptomycin/penicillin (1%, Solarbio).

### Cell transfection

Lentivirus vectors carrying short hairpin (sh) RNA targeting circ_0061395 (sh-circ_0061395) or matching negative control (NC) (sh-NC) were purchased from GenePharma (Shanghai, China). Small interference (si) RNA targeting circ_0061395 (si-circ_0061395) and corresponding NC (si-NC) were synthesized from GenePharma. MiR-877-5p mimic, miR-877-5p inhibitor, and their matching NCs (miRNA NC and inhibitor NC) were purchased from RiboBio (Guangzhou, China). For the pcDNA3.1-PIK3R3 (pc-PIK3R3) plasmid, the full-length sequence of PIK3R3 was cloned into the empty pcDNA3.1 vector (pc-NC) (Life Technologies, Grand Island, NY, USA). Transient transfection was performed using Lipofectamine 3000 reagent (Invitrogen, Carlsbad, CA, USA).

### Exosome isolation

Exosomes were isolated from the serum of HCC patients and healthy controls using the ExoQuick exosome precipitation solution (System Biosciences, Mountain View, CA, USA). In short, the mixture of serum and ExoQuick exosome precipitation solution was stored at 4 °C for 24 h. Thereafter, the sample was centrifuged at 1500×*g* for 30 min to obtain exosome pellets. Then, these exosome pellets were suspended in phosphate-buffered saline for subsequent analysis. For nanoparticle-tracking analysis (NTA), the number and size of exosomes were tracked using NanoSight NS 300 system (NanoSight, Malvern, UK) and particle tracking software (NanoSight).

### Transmission electron microscopy (TEM)

The exosome suspension was placed on a special copper mesh of the H-7650 electron microscope (Hitachi, Tokyo, Japan), followed by negative staining with 2% phosphotungstic acid (20 μL) for 10 min. The morphology of exosomes was observed by the H-7650 electron microscope (Hitachi) at 100 kV.

### Western blotting

Total protein was extracted using the RIPA lysis buffer (Solarbio). Then, the extracted total protein (30 μg) was subjected to sodium dodecyl sulfate–polyacrylamide gel electrophoresis (12%, SDS-PAGE), followed by transferring onto the polyvinylidene fluoride (PVDF, Millipore, Billerica, MA, USA) membrane. After blocking with Tris Buffered Saline Tween (TBST) buffer containing 5% non-fat milk, the PVDF membrane was incubated with primary antibodies anti-CD63 (K007602P, 1:500, Solarbio), anti-CD81 (K000385P, 1:500, Solarbio), anti-PIK3R3 (K008995P, 1:1000, Solarbio), anti-GAPDH (K106389P, 1:5000, Solarbio) at 4˚C overnight. Next, the PVDF membrane was incubated with goat anti-rabbit IgG (SE134, 1:2000, Solarbio). Finally, the immunoblot was visualized through enhanced chemiluminescence solution (Solarbio).

### Quantitative real-time polymerase chain reaction (qRT-PCR)

Nuclear and cytoplasmic RNA of HCC cells were extracted with the PARIS kit (Life Technologies). RNAiso Plus (TaKaRa, Tokyo, Japan) or miRNeasy Serum/Plasma Kit (Qiagen, Valencia, CA, USA) was utilized to isolate total RNA from sera, tissue samples, and cells. For complementary DNA generation, total RNA was reversely transcribed with Moloney Murine Leukemia Virus (M-MLV) First Strand Kit (Life Technologies) or commercial miR reverse transcription PCR kit (RiboBio). The synthesized complementary DNA was used for qPCR with the SYBR Green PCR Master Mix (Bio-Rad, Hercules, CA, USA). The primers were used as follows: circ_0061395 (F: 5′-AGCGCTGTCGCAAGAGAAAA-3′; R: 5′-CTTTAACTGTCACCAGCTTCTCAA-3′), glyceraldehyde-3-phosphate dehydrogenase (GAPDH) (F: 5′-GACTCCACTCACGGCAAATTCA-3′; R: 5′-TCGCTCCTGGAAGATGGTGAT-3′), BACH1 (F: 5′-TCTGAGTGAGAACTCGGTTTTTG-3′; R: 5′-CGCTGGTCATTAAGGCTGAGTAA-3′), PIK3R3 (F: 5′-CTTTGCGGAAGGGAGGCAATA-3′; R: 5′-ACCACGGAATTAAATGTCAGAGG-3′), miR-877-5p (F: 5′-GTAGAGGAGATGGCGCAGGG-3′; R: 5′-CAGTGCGTGTCGTGGAGT-3′), and U6 small nuclear RNA (U6) (F: 5′-GCTCGCTTCGGCAGCACA-3′; R: 5′-GAGGTATTCGCACCAGAGGA-3′). Relative expression levels were figured with the 2^−ΔΔCt^ method, and GAPDH or U6 was used as an internal control.

### Cell viability assay

After a specific transfection, HCC cells (1 × 10^3^ cells/well) were seeded to a 96-well plate. After culturing for 48 h, the cell counting kit-8 (CCK-8) solution (10 μL, Dojindo, Kumamoto, Japan) was added to each well and incubated for 1 h. The absorbance at 450 nm was measured by the Microplate Reader (Bio-Rad).

### Plate clone assay

Transfected HCC cells (1 × 10^2^ cells/well) were seeded into 6-well plates. After culture for 2 weeks, the cells were washed with phosphate buffer solution and then fixed with paraformaldehyde (4%, Solarbio). Thereafter, the cells were stained with crystal violet (0.5%, Solarbio). Finally, the number of cell colonies (> 50 cells) were counted and photographed with an inverted microscope (Nikon, Tokyo, Japan).

### Transwell assay

The invasion and migration abilities of HCC cells were analyzed by transwell chambers with an 8 μm pore membrane (Costar, Cambridge, MA, USA). For the invasion assay, the transwell chamber was pre-covered with Matrigel (Sigma). In short, the upper chamber was added with serum-free DMEM or RPMI-1640 medium (200 μL) containing HCC cells (1 × 10^5^ cells), and the lower chamber was added with DMEM or RPMI-1640 medium (600 μL) containing FBS (10%). After 24 h incubation, the migrating and invading cells were fixed with paraformaldehyde (4%, Solarbio) and then stained with crystal violet (0.5%, Solarbio). The number of the migrating and invading cells was figured by the inverted microscope (Nikon) (magnification, 100 ×).

### Flow cytometry assay

Transfected HCC cells were harvested and treated with trypsin, followed by washing with phosphate buffer solution. For the cell cycle assay, these cells were fixed with ethanol (70%) and then stained with propidium iodide (PI)/RNase solution (Sigma). For the cell apoptosis analysis, these cells were stained with the Annexin V-fluorescein isothiocyanate (FITC)/PI Apoptosis Detection Kit (BD Biosciences, San Jose, CA, USA). The cell distribution was analyzed by using a FACScan flow cytometry (BD Biosciences) with a FACS Diva Software (BD Biosciences). The data of the cell cycle experiment and the cell apoptosis experiment were presented in Additional file 1 and Additional file 2, respectively.

### Dual-luciferase reporter assay

The binding sites of circ_0061395 or PIK3R3 in miR-877-5p were predicted with the CircInteractome (https://circinteractome.irp.nia.nih.gov/mirna_target_sites.html) or starBase (http://starbase.sysu.edu.cn/) databases. For luciferase reporter plasmids generation, the fragment of WT (wild type) -circ_0061395), MUT (mutant)-circ_0061395, WT-PIK3R3-3′UTR, or MUT-PIK3R3-3′UTR was synthesized and then inserted into the psiCHECK-2 vectors (Promega, Madison, WI, USA). Next, HCC cells were a transfected with a luciferase reporter plasmid and miRNA NC or miR-877-5p mimic. After transfection for 48 h, the firefly and Renilla luciferase intensities were assessed with the luciferase reporter assay system (Promega). The relative luciferase intensity was evaluated by normalizing the firefly luminescence to Renilla luminescence.

### RNA immunoprecipitation (RIP) assay

The specific binding between circ_0061395 and miR-877-5p was analyzed using the Magna RIP Kit (Millipore, Billerica, MA, USA). Briefly, HCC cells (80%-90% confluence) were lysed in RIP lysis buffer. After that, the extract (100 μL) was incubated with immunoprecipitation buffer containing magnetic beads conjugated with Anti-Ago_2_ (ab32381, Abcam, Cambridge, MA, USA) or Anti-IgG (ab133470, Abcam). After RNA isolation, the levels of circ_0061395 and miR-877-5p were analyzed with qRT-PCR.

### Xenograft assay

The protocols of xenograft assay were authorized by the Animal Ethics Committee of the Second Hospital of Jilin University. For xenograft assay, Huh7 cells (1 × 10^6^ cells/200 μL) with sh-circ_0061395 or sh-NC were subcutaneously injected into the right flank of BALB/c nude mice (6 weeks old, Huafukang Bioscience, Beijing, China) (5 mice/group). Tumor volume was measured once a week with a digital caliper. Until day 28, these mice were sacrificed by cervical dislocation under isoflurane (5%) for subsequent analysis. Tumor volume was calculated in accordance with the following equation: Volume = (length × width^2^)/2. All mice were kept under specific pathogen-free conditions. For stable knockdown of circ_0061395, Huh7 cells were transduced with lentiviral particles carrying sh-circ_0061395.

### Immunohistochemical (IHC) staining

The immunohistochemical staining was performed as previously described [22]. The paraffin-embedded tissue was incubated with an antibody against Ki67 (ab16667, 1:200, Abcam).

### Statistical analysis

The experiments in vitro were repeated at last 3 times and each experiment was carried out in triplicate. The original data of this study was displayed in Additional file 3. Statistical analysis was implemented with SPSS 20.0 software (SPSS, Chicago, IL, USA). Data were displayed as the mean ± standard deviation. These data followed a normal distribution using the Kolmogorov–Smirnov test. The homogeneity of variance between 2 groups was assessed by F-test. The difference between 2 groups was evaluated with paired or unpaired Student’s *t* test. The differences among 3 or more groups were evaluated by one-way variance analysis (ANOVA) with Turkey’s post hoc test. Correlation between circ_0061395 expression and clinicopathological parameters of HCC patients were evaluated by Chi-square test. *P* < 0.05 was considered statistically significant.

## Results

### Circ_0061395 was upregulated in serum exosomes of HCC patients

**Fig. 1:**
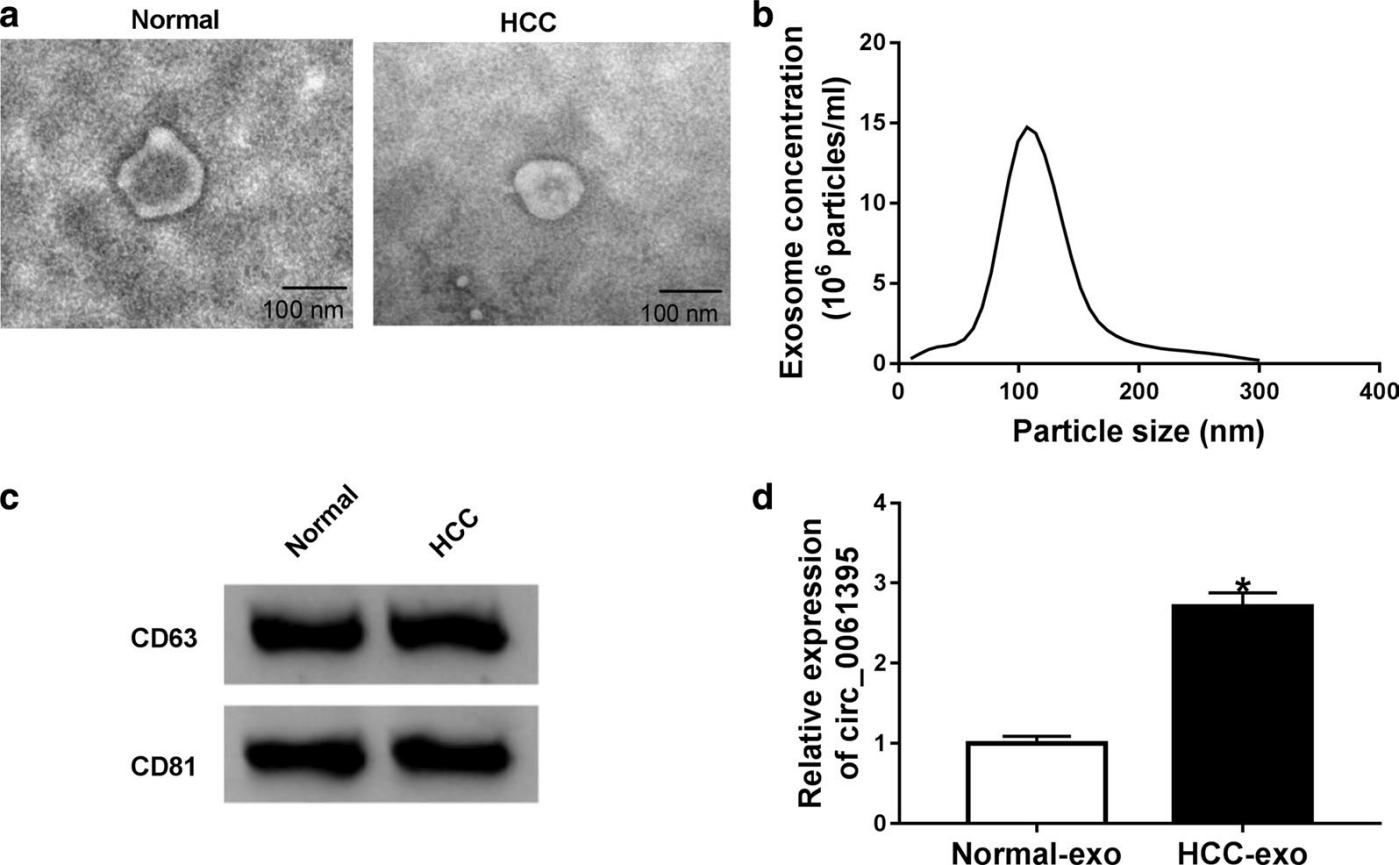
Circ_0061395 was upregulated in serum exosomes of HCC patients. **a** The morphology of exosomes from the serum of HCC patients and healthy controls were observed under TEM. **b** NTA analysis revealed the size of exosomes from the serum of HCC patients and healthy controls. **c** Western blotting was executed to detect the exosomal markers CD63 and CD81 in serum exosomes of HCC patients and healthy controls. **d** The expression of circ_0061395 in serum exosomes of HCC patients and healthy controls was analyzed by qRT-PCR. *P < 0.05

At the outset, we extracted exosomes from the serum of HCC patients and healthy controls. TEM presented that the serum exosomes of HCC patients and healthy controls were round (Fig. 1a). NTA analysis displayed that the size of exosomes was about 100 nm (Fig. 1b). Western blotting showed the presence of CD63 and CD81 (exosome markers) present in exosomes derived from the serum of HCC patients and healthy controls (Fig. 1c). QRT-PCR exhibited that circ_0061395 expression was observably increased in exosomes derived from serum of HCC patients compared to exosomes derived from healthy control serum (Fig. 1d). These results indicated that high circ_0061395 expression in serum-derived exosomes might be involved in HCC development.

### Circ_0061395 expression was apparently elevated in HCC

**Fig. 2:**
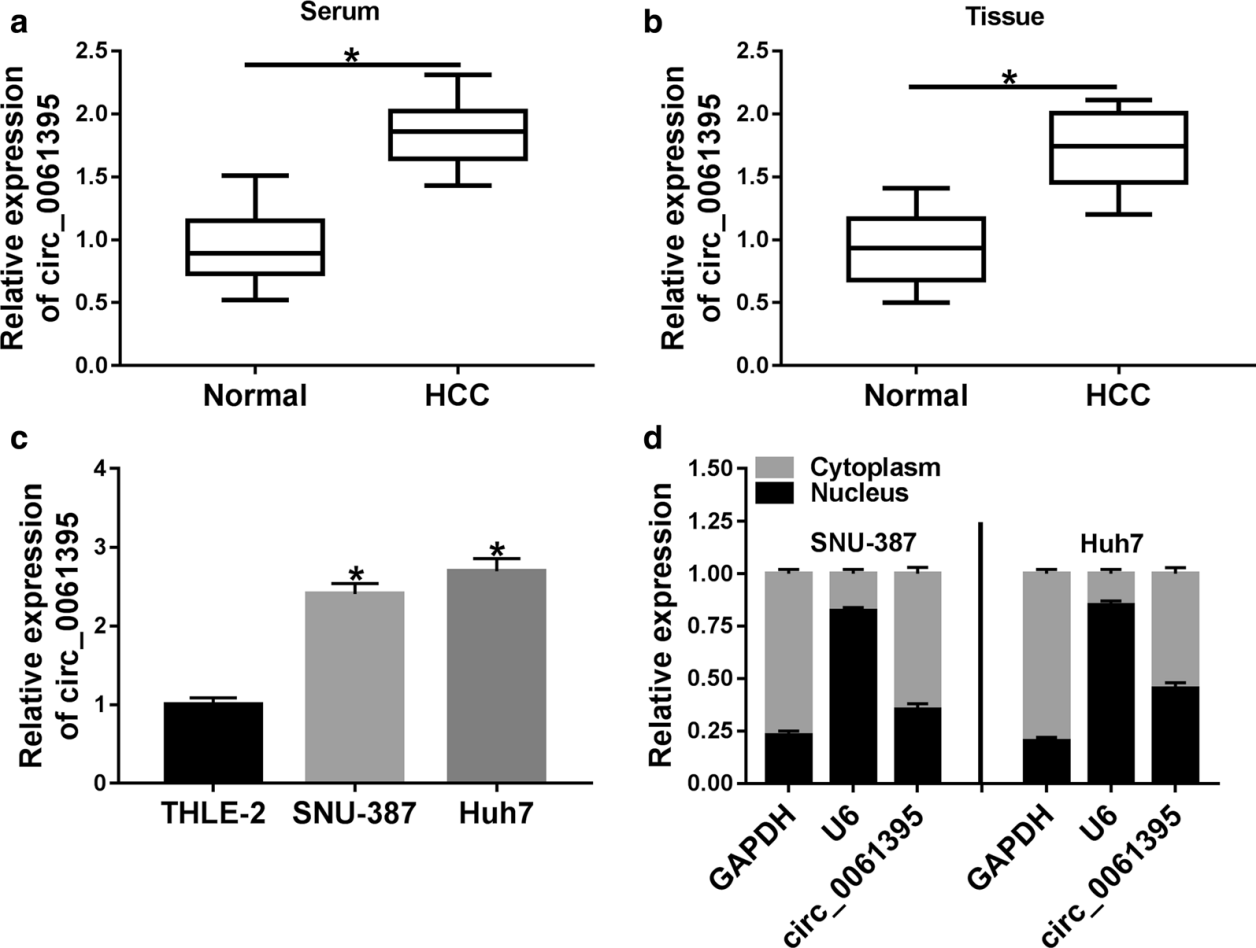
Circ_0061395 was highly expressed in HCC. **a**–**c** QRT-PCR was performed to assess the expression of circ_0061395 in HCC serum, tissues, and cells. **d** The levels of circ_0061395, U6, and GAPDH in the cytoplasm and nucleus of SNU-387 and Huh7 cells were analyzed by qRT-PCR. *P < 0.05

To investigate the biological function of circ_0061395 in HCC, we detected circ_0061395 expression in the serum of 30 HCC patients and 30 healthy controls. QRT-PCR presented that circ_0061395 was higher expression in the serum of HCC patients than that the healthy controls (Fig. 2a). Also, circ_0061395 expression was elevated in HCC tissues when compared to the adjacent non-tumor tissues (Fig. 2b). Furthermore, circ_0061395 expression was associated with the tumor size, TNM stages, and lymphatic metastasis of HCC patients (Table 1). We also observed that circ_0061395 expression was elevated in HCC cells (SNU-387 and Huh7) than that in THLE-2 cells (Fig. 2c). Nucleus and cytoplasm separation experiment and qRT-PCR exhibited that circ_0061395 was mainly located in the cytoplasm of SNU-387 and Huh7 cells (Fig. 2d). These data indicated that high circ_0061395 expression might be associated with HCC advancement.

**Table 1:** Correlation between circ_0061395 expression and clinicopathological parameters of HCC patients

Parameter	Case	Circ_0061395 expression		*P* value^a^
		Low (n=12)	High (n=18)	
Age (years)				0.722
≤ 60	14	5	9	
> 60	16	7	9	
Gender				0.139
Female	10	6	4	
Male	20	6	14	
Tumor size				0.008*
≤ 5 cm	13	9	4	
> 5 cm	17	3	14	
TNM stages				0.009*
I–II	11	8	3	
III	19	4	15	
Lymphatic metastasis				0.0001*
Negative	12	10	2	
Positive	18	2	16	
Vascular invasion				0.457
Absent	16	5	11	
Present	14	7	7	

*HCC* hepatocellular carcinoma, *TNM* tumor-node-metastasis

*P < 0.05

^a^Chi-square test

### Circ_0061395 knockdown constrained malignant behaviors of HCC cells

**Fig. 3:**
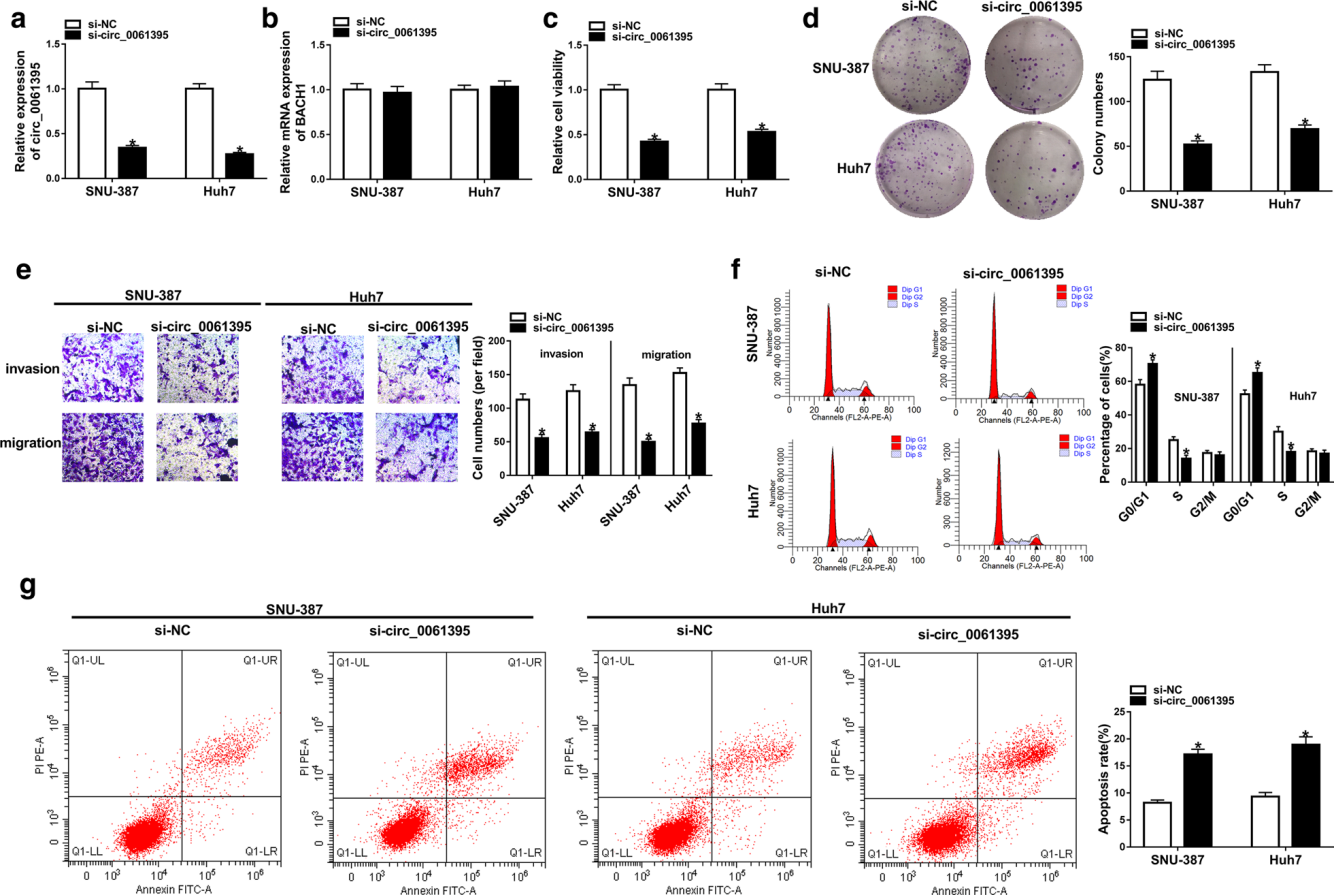
Inhibition of circ_0061395 reduced the malignancy of HCC cells. **a**–**g** SNU-387 and Huh7 cells were transfected with si-NC or si-circ_0061395. **a**, **b** The expression levels of circ_0061395 and BACH1 mRNA in SNU-387 and Huh7 cells were examined by qRT-PCR. **c**–**g** The viability, colony formation, invasion, migration, cell cycle progression, and apoptosis of SNU-387 and Huh7 cells were determined by CCK-8, plate clone, transwell, or flow cytometry assays. *P < 0.05

Subsequently, we investigated the biological role of circ_0061395 in HCC through the loss-of-function experiments. The knockdown efficiency of si-circ_0061395 in SNU-387 and Huh7 cells was exhibited in Fig. 3a. The transfection of si-circ_0061395 had no overt influence on the expression of BACH1 mRNA (Fig. 3b). CCK-8 and plate clone assays indicated that circ_0061395 silencing reduced the proliferation of SNU-387 and Huh7 cells (Fig. 3c, d). Transwell assay exhibited that circ_0061395 knockdown impeded the invasion and migration of SNU-387 and Huh7 cells (Fig. 3e). Flow cytometry assay displayed that decreased circ_0061395 expression induced cell cycle arrest and apoptosis in SNU-387 and Huh7 cells (Fig. 3f, g). Taken together, these data indicated that circ_0061395 knockdown could reduce the malignancy of HCC cells.

### Circ_0061395 was identified as a sponge for miR-877-5p in HCC cells

**Fig. 4:**
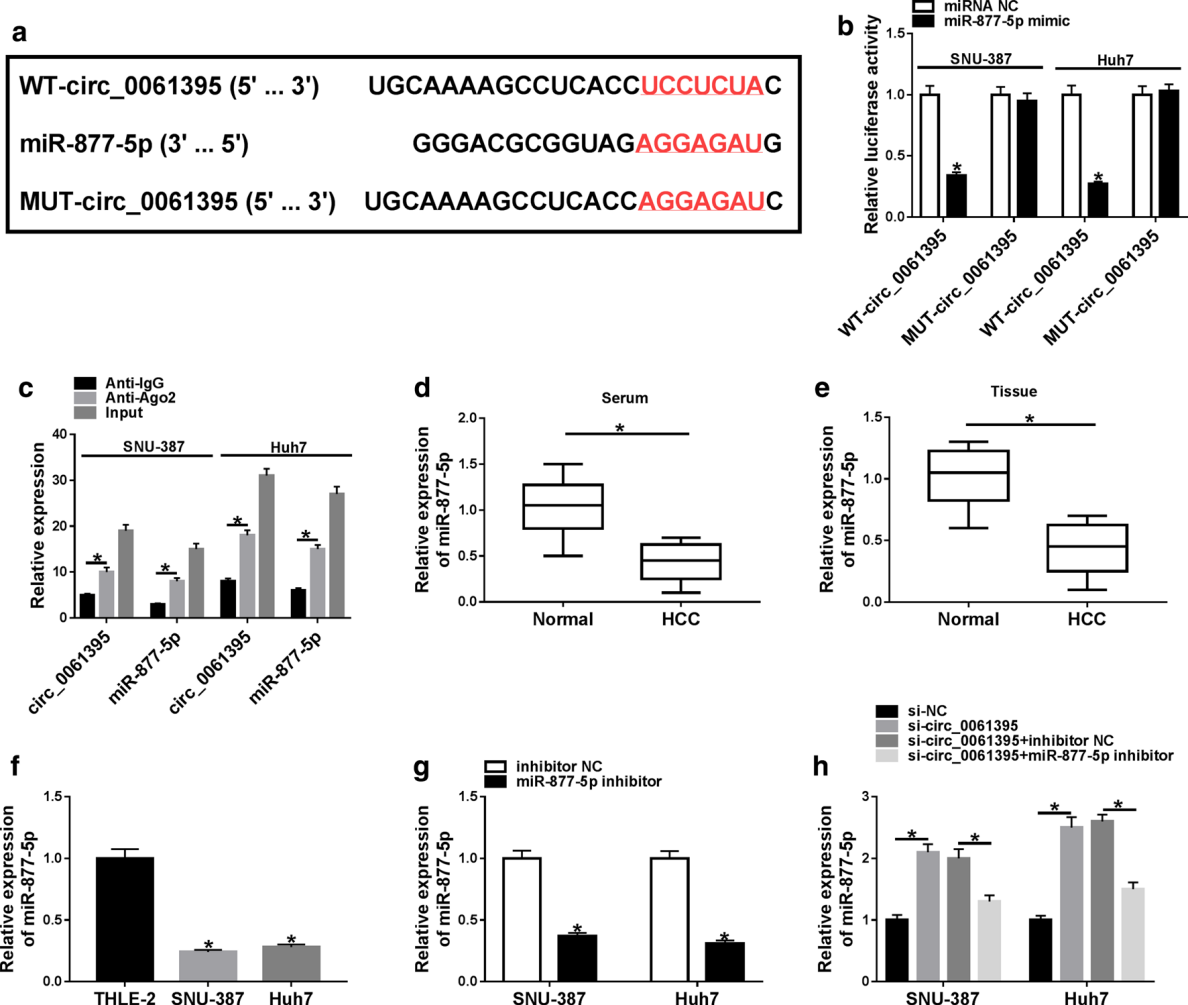
Circ_0061395 served as a sponge for miR-877-5p in HCC cells. **a** The complementary binding sites between circ_0061395 and miR-877-5p were predicted by the CircInteractome database. **b** Dual luciferase reporter assay was performed to assess the luciferase activity in SNU-387 and Huh7 cells cotransfected with luciferase plasmids containing MUT-circ_0061395 or WT-circ_0061395 and miRNA NC or miR-877-5p mimic. **c** After RIP assay, the levels of circ_0061395 and miR-877-5p were examined by qRT-PCR. **d**–**f** The expression of miR-877-5p in HCC serum, tissues, and cells was detected by qRT-PCR. **g** After inhibitor NC or miR-877-5p inhibitor transfection, the expression of miR-877-5p in SNU-387 and Huh7 was examined by qRT-PCR. (H) The expression of miR-877-5p in SNU-387 and Huh7 cells transfected with si-NC, si-circ_0061395, si-circ_0061395 + inhibitor NC, or si-circ_0061395 + miR-877-5p inhibitor was analyzed by qRT-PCR. *P < 0.05

To investigate the underlying molecular mechanism of circ_0061395 in HCC, we predicted miRs which could bind to circ_0061395 using the CircInteractome database. As exhibited in Fig. 4a, circ_0061395 might be a sponge of miR-877-5p. Dual-luciferase reporter assay exhibited that miR-877-5p mimic could reduce the luciferase activity of the luciferase plasmid with WT-circ_0061395 in SNU-387 and Huh7 cells, while the luciferase activity of the luciferase plasmid containing MUT-circ_0061395 did not change (Fig. 4b). RIP assay presented that circ_0061395 and miR-877-5p were enriched in the anti-Ago2 group compared to the Anti-IgG group (Fig. 4b). Moreover, miR-877-5p expression was reduced in HCC serum, tissues, and cells (Fig. 4d–f). The knockdown efficiency of miR-877-5p inhibitor in in SNU-387 and Huh7 cells was verified by qRT-PCR (Fig. 4g). Additionally, circ_0061395 silencing elevated miR-877-5p expression in SNU-387 and Huh7 cells, but this increase was restored by miR-877-5p inhibitor (Fig. 4h). These data suggested that circ_0061395 acted as a sponge for miR-877-5p in HCC cells.

### Circ_0061395 regulated malignant behaviors of HCC cells by sponging miR-877-5p

**Fig. 5:**
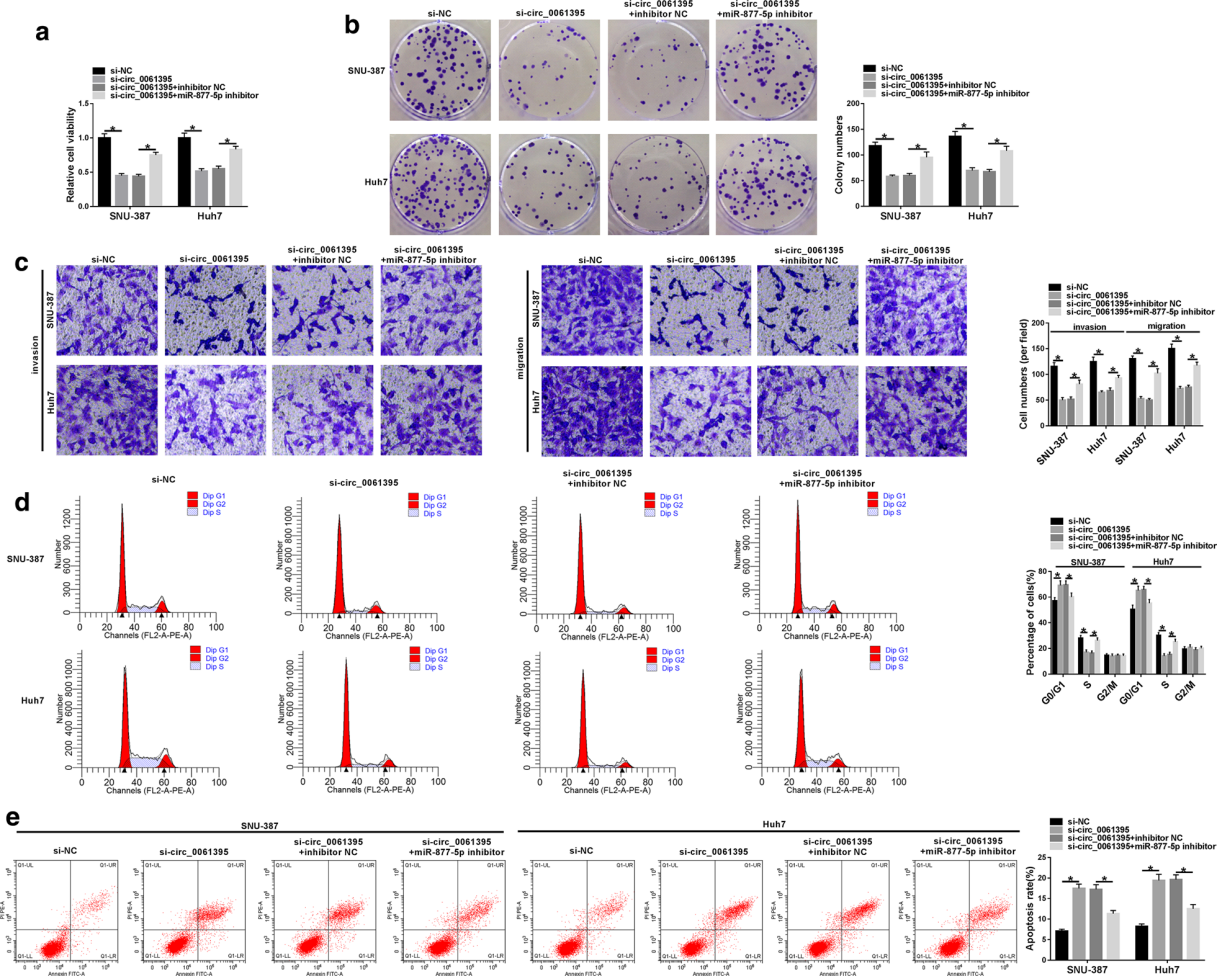
Circ_0061395 exerted its role in HCC cells via sponging miR-877-5p. **a**–**e** The viability, colony formation, invasion, migration, cell cycle progression, and apoptosis of SNU-387 and Huh7 cells transfected with si-NC, si-circ_0061395, si-circ_0061395 + inhibitor NC, or si-circ_0061395 + miR-877-5p inhibitor were assessed by CCK-8, plate clone, transwell, or flow cytometry assays. *P < 0.05

In consideration of the above findings, we explored whether circ_0061395 facilitated malignant behaviors of HCC cells through adsorbing miR-877-5p. The results exhibited that miR-877-5p inhibitor overturned the repressive influence of circ_0061395 silencing on proliferation, invasion, and migration of SNU-387 and Huh7 cells (Fig. 5a–c). Flow cytometry assay exhibited that miR-877-5p silencing offset the promoting influence of circ_0061395 inhibition on cell cycle arrest and apoptosis in SNU-387 and Huh7 cells (Fig. 5d, e). In sum, these findings suggested that circ_0061395 regulated the malignancy of HCC cells via sponging miR-877-5p in HCC cells.

### MiR-877-5p repressed the malignancy of HCC cells via targeting PIK3R3

**Fig. 6:**
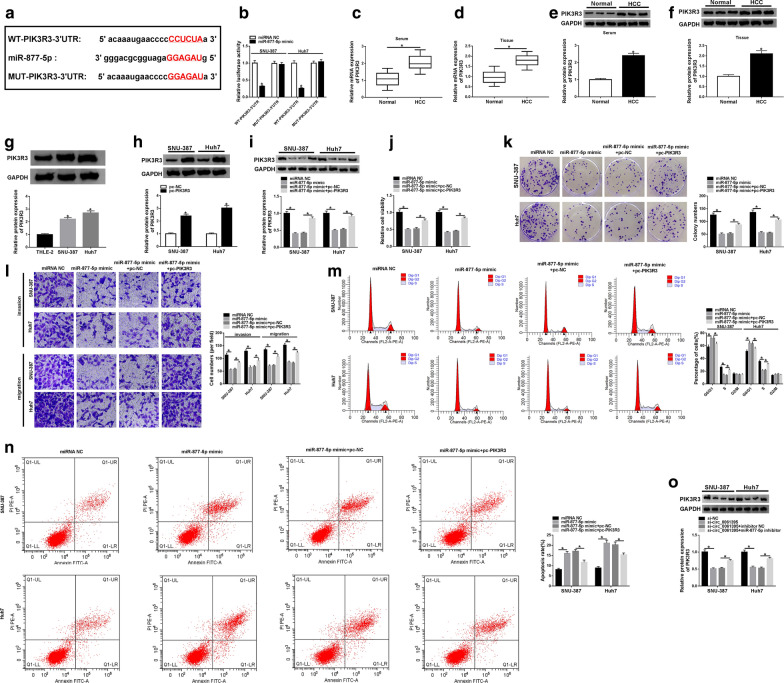
Circ_0061395 regulated PIK3R3 expression through sponging miR-877-5p in HCC cells. **a** The binding sites of PIK3R3 in miR-877-5p were predicted by the starBase database. **b** Dual-luciferase reporter assay was executed to determine the binding sites between PIK3R3 and miR-877-5p in SNU-387 and Huh7 cells. **c**–**g** QRT-PCR and western blotting were employed to assess the levels of PIK3R3 mRNA and/or protein in HCC serum, tissues, and cells. **h** After pc-NC or pc-PIK3R3 transfection, the level of PIK3R3 protein in SNU-387 and Huh7 cells was detected with western blotting. (I-N) SNU-387 and Huh7 cells were transfected with miRNA NC, miR-877-5p mimic, miR-877-5p mimic + pc-NC, or miR-877-5p mimic + pc-PIK3R3. **i** The level of PIK3R3 protein in SNU-387 and Huh7 cells was analyzed by western blotting. **j**–**n** The viability, colony formation, invasion, migration, cell cycle progression, and apoptosis of SNU-387 and Huh7 cells were analyzed by CCK-8, plate clone, transwell, or flow cytometry assays. **o** The level of PIK3R3 protein in SNU-387 and Huh7 cells transfected with si-NC, si-circ_0061395, si-circ_0061395 + inhibitor NC, or si-circ_0061395 + miR-877-5p inhibitor was measured by western blotting. *P < 0.05

Next, we explored the downstream targets of miR-877-5p. Online bioinformatic prediction (starBase) revealed that PIK3R3 might be a downstream target of miR-877-5p (Fig. 6a). Moreover, miR-877-5p mimic could reduce the luciferase intensity of the luciferase vector containing WT-PIK3R3-3′UTR in SNU-387 and Huh7 cells but not the luciferase vector containing MUT-PIK3R3-3′UTR (Fig. 6b). Also, the levels of PIK3R3 mRNA and protein were observably elevated in the serum and tumor tissues of HCC patients (Fig. 6c–f). There was a marked upregulation of PIK3R3 protein in HCC cells (SNU-387 and Huh7 cells) (Fig. 6g). After pc-PIK3R3 transfection, the level of PIK3R3 protein was dramatically elevated in SNU-387 and Huh7 cells (Fig. 6h). Moreover, miR-877-5p mimic decreased the level of PIK3R3 protein in SNU-387 and Huh7 cells, while this influence was restored by forcing PIK3R3 expression (Fig. 6i). Then, we verified whether miR-877-5p regulated the malignancy of HCC cells through targeting PIK3R3. The overexpression of PIK3R3 counteracted the inhibitory impact of miR-877-5p mimic on proliferation, invasion, and migration of SNU-387 and Huh7 cells (Fig. 6j–l). Also, PIK3R3 overexpression reversed miR-877-5p mimic-induced cell cycle arrest and apoptosis in SNU-387 and Huh7 cells (Fig. 6m, n). In addition, circ_0061395 silencing reduced the level of PIK3R3 protein in SNU-387 and Huh7 cells, but this decrease was overturned after miR-877-5p inhibitor transfection (Fig. 6o). Overall, these results indicated that circ_0061395 regulated malignant behaviors of HCC cells by the miR-877-5p/PIK3R3 pathway.

### Circ_0061395 knockdown decreased tumor growth in vivo

**Fig. 7:**
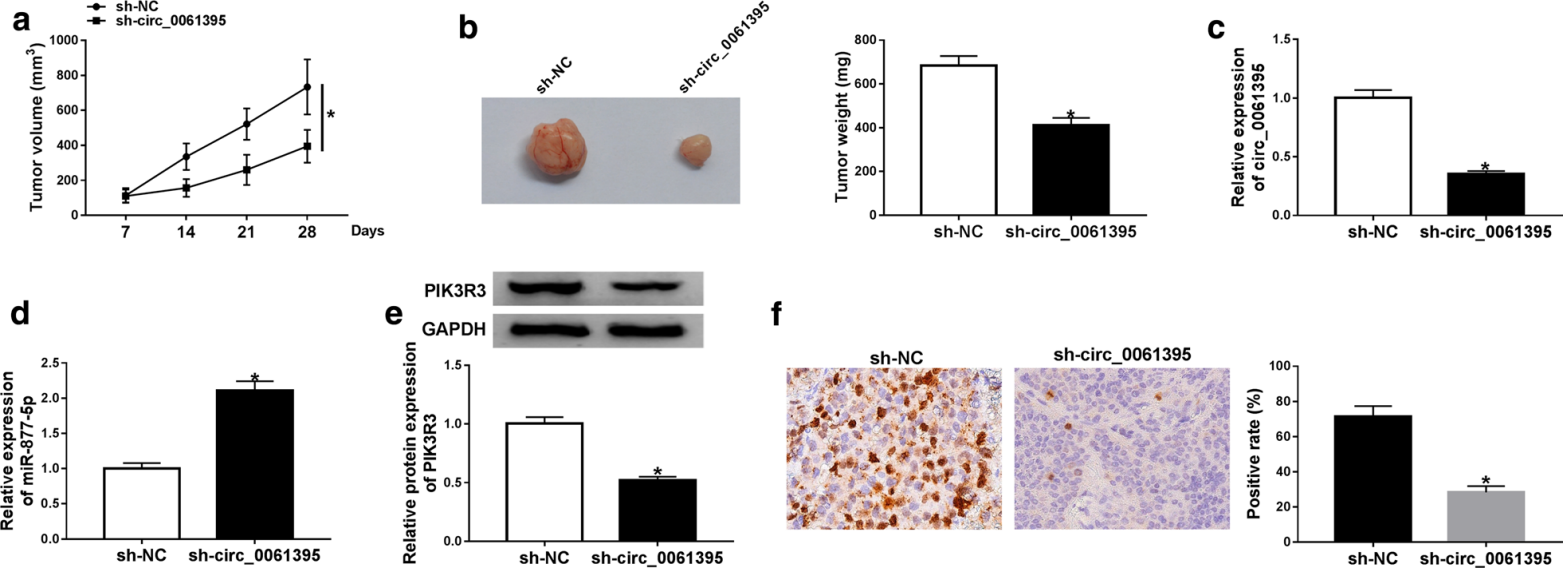
Circ_0061395 silencing decreased tumor growth in vivo. **a**, **b** Tumor volume and weight of mice in the sh-circ_0061395 and sh-NC groups. **c**–**e** QRT-PCR and western blotting were performed to assess the levels of circ_0061395, miR-877-5p, and PIK3R3 protein in mice tumor tissues of the sh-circ_0061395 and sh-NC groups. **f** IHC analysis of Ki67 expression in mice tumor tissues of the sh-circ_0061395 and sh-NC groups. *P < 0.05

To verify the function of circ_0061395 in HCC, we performed xenograft assay through injecting Huh7 cells carrying sh-circ_0061395 or sh-NC into nude mice. We observed that tumor volume and weight were reduced in mice of the sh-circ_0061395 group relative to the sh-NC group (Fig. 7a, b). Moreover, circ_0061395 was downregulated while miR-877-5p was upregulated in mice tumor tissues of the sh-circ_0061395 group when compared with the sh-NC group (Fig. 7c, d). Also, the level of PIK3R3 protein was reduced in mice tumor tissues of the sh-circ_0061395 group (Fig. 7e). IHC staining displayed that the expression of Ki67 was decreased in mice tumor tissues of the sh-circ_0061395 group in contrast to the sh-NC group (Fig. 7f). These data suggested that circ_0061395 inhibition could decrease tumor growth in vivo.

## Discussion

Increasing researches have proved that circRNAs exert a vital role in tumor growth [23]. More and more circRNAs have been revealed to exert carcinogenic or tumor-suppressive influence on HCC based on the development of sequencing technology. Studies have reported that circRNA circ_104718 [24], circRNA circ_CDR1AS [25], and circRNA circ_RHOT1 [26] play a cancerigenic role in HCC, but circRNA circ_5692 [27] and circRNA circ_TRIM33-12 [28] exert anti-tumor role in HCC. In this study, circ_0061395 expression was elevated in HCC tissues, serum, and cells. Moreover, circ_0061395 inhibition reduced tumor growth in vivo and impeded cell viability, colony formation, invasion, migration, and induced cell cycle arrest and apoptosis in HCC cells in vitro. Liu et al. discovered that circ_0061395 facilitated HCC development via inhibiting P27 expression via binding to HuR [10]. These results manifested that circ_0061395 could accelerate the advancement of HCC.

Studies have proved that the abnormal expression of miRs is related to the development of a series of cancers, including HCC [29, 30]. Also, circRNAs take part in the advancement of a range of tumors by sponging miRs [31]. MiR-877-5p had been proved to exert a repressive role in several cancers. Liang et al. uncovered that lncRNA DSCAM-AS1 inhibition elevated the expression of miR-877-5p, thereby repressing invasion, migration, and proliferation of cervical cancer cells [13]. Report of Xie et al. revealed that lncRNA TRG-AS1 accelerated proliferation of glioblastoma through elevating SUZ12 expression by adsorbing miR-877-5p [14]. In HCC, miR-877-5p could decrease cancer cell invasion, migration, and growth via targeting CDK14 [15]. Herein, we discovered that circ_0061395 acted as a sponge for miR-877-5p. Downregulation of miR-877-5p overturned circ_0061395 silencing-mediated the malignancy of HCC cells. Therefore, we inferred that circ_0061395 accelerated the malignancy of HCC cells via sponging miR-877-5p.

PIK3R3 is a known oncogene, which accelerated a range of tumors development, such as cervical cancer [16], ovarian cancer [17], glioma [19], and colorectal cancer [18]. In HCC, miR-601 [32], miR-1287 [20], and miR-132 [21] could repress HCC progression via targeting PIK3R3. Furthermore, lncRNA LINC00160 knockdown reduced drug resistance and autophagy by downregulating PIK3R3 by sponging miR-132 in HCC [21]. Herein, PIK3R3 acted as a downstream target for miR-877-5p. PIK3R3 overexpression abolished the repressive impact of miR-877-5p mimic on the malignancy of HCC cells. Furthermore, circ_0061395 regulated PIK3R3 expression via adsorbing miR-877-5p. Overall, we concluded that circ_0061395 regulated the malignancy of HCC cells via regulating the miR-877-5p/PIK3R3 axis.

Exosomes, a type of microvesicle (diameter about 40–100 nm), have been revealed to exert a vital role in HCC advancement [33]. Exosomes contain a variety of circRNAs, miRs, lipids, and proteins, which act as information transmitters in intercellular communication [34, 35]. Previous study revealed that that the exosomal circRNA circ_100383 derived from highly metastatic HCC cells contributed to HCC metastasis through elevating angiogenesis and invasiveness [36]. Zhang et al. indicated that adipocytes-derived exosomal circRNA circ_DB accelerated HCC progression via activation of the USP7/cyclin A2 pathway via sponging miR-34a [37]. In the current study, we discovered that circ_0061395 was elevated in serum exosomes of HCC patients. Unfortunately, we did not explore whether the exosomal circ_0061395 derived from the serum of HCC patients affected the tumorigenesis and advancement of HCC, which can be investigated in the future (Additional files 1, 2 and 3).

## Conclusion

In conclusion, circ_0061395 promoted the malignancy of HCC cells via elevating PIK3R3 expression through competitively binding to miR-877-5p, which offered that circ_0061395 might be a target for HCC treatment.

## Supplementary Information

**Additional file 1.** The distribution of cells at each stage of all cell cycle experiments.

**Additional file 2.** The percentage of cells in each quadrant in all apoptosis experiments.

**Additional file 3**. Raw data from three repeated experiments.

## Data Availability

The analyzed data sets generated during the present study are available from the corresponding author on reasonable request.

## Abbreviations

circ_0061395: Circular RNA hsa_circ_0061395
HCC: Hepatocellular carcinoma
TEM: Transmission electron microscope
NTA: Nanoparticle-tracking analysis
PIK3R3: Phosphoinositide-3-kinase regulatory subunit 3
qRT-PCR: Quantitative real time polymerase chain reaction
CCK-8: Cell counting kit-8
RIP: RNA immunoprecipitation
