# Supplementary material for: Circular RNA hsa_circ_0061395 accelerates hepatocellular carcinoma progression via regulation of the miR-877-5p/PIK3R3 axis
Source: Cancer Cell Int. 2021 Jan 6;21:10. doi: 10.1186/s12935-020-01695-w (PMC7788978; doi:10.1186/s12935-020-01695-w)
Supplement: Supplementary file 2 — Additional file 2. The percentage of cells in each quadrant in all apoptosis experiments. [file 12935_2020_1695_MOESM2_ESM.doc]

Fig3G

| **SNU-387** | Q1-UR | Q1-UL | Q1-LL | Q1-LR |
| --- | --- | --- | --- | --- |
| Si-NC | 7.19% | 0.77% | 91.25% | 0.79% |
| Si-circ_0061395 | 14.67% | 2.43% | 81.66% | 1.24% |

| **Huh7** | Q1-UR | Q1-UL | Q1-LL | Q1-LR |
| --- | --- | --- | --- | --- |
| Si-NC | 8.77% | 1.21% | 88.80% | 1.22% |
| Si-circ_0061395 | 17.47% | 1.17% | 79.40% | 1.97% |

Fig5E

| **SNU-387** | Q1-UR | Q1-UL | Q1-LL | Q1-LR |
| --- | --- | --- | --- | --- |
| Si-NC | 4.21% | 0.79% | 94.32% | 0.67% |
| Si-circ_0061395 | 15.04% | 2.76% | 81.00% | 1.20% |
| Si-circ_0061395+inhibitor NC | 16.72% | 1.57% | 80.31% | 1.40% |
| Si-circ_0061395+miR-877-5p inhibitor | 11.42% | 1.90% | 85.46% | 1.22% |

| **Huh7** | Q1-UR | Q1-UL | Q1-LL | Q1-LR |
| --- | --- | --- | --- | --- |
| Si-NC | 7.09% | 1.07% | 90.96% | 0.88% |
| Si-circ_0061395 | 18.73% | 1.28% | 78.72% | 1.26% |
| Si-circ_0061395+inhibitor NC | 18.60% | 1.66% | 78.58% | 1.16% |
| Si-circ_0061395+miR-877-5p inhibitor | 12.28% | 1.35% | 85.46% | 0.91% |

Fig6N

| **Huh7** | Q1-UR | Q1-UL | Q1-LL | Q1-LR |
| --- | --- | --- | --- | --- |
| miRNA NC | 7.09% | 0.81% | 91.40% | 0.70% |
| miR-877-5p mimic | 14.93% | 2.53% | 81.34% | 1.20% |
| miR-877-5p+pc-NC | 16.72% | 1.57% | 80.14% | 1.57% |
| miR-877-5p+pc-PIK3R3 | 11.24% | 2.16% | 85.47% | 1.13% |

| **Huh7** | Q1-UR | Q1-UL | Q1-LL | Q1-LR |
| --- | --- | --- | --- | --- |
| miRNA NC | 7.99% | 1.34% | 89.69% | 0.98% |
| miR-877-5p mimic | 18.41% | 1.04% | 77.78% | 2.77% |
| miR-877-5p+pc-NC | 17.92% | 2.16% | 78.59% | 1.34% |
| miR-877-5p+pc-PIK3R3 | 13.65% | 1.25% | 84.00% | 1.10% |
