# Supplementary material for: Circular RNA hsa_circ_0061395 accelerates hepatocellular carcinoma progression via regulation of the miR-877-5p/PIK3R3 axis
Source: Cancer Cell Int. 2021 Jan 6;21:10. doi: 10.1186/s12935-020-01695-w (PMC7788978; doi:10.1186/s12935-020-01695-w)
Supplement: Supplementary file 3 — Additional file 3. Raw data from three repeated experiments. [file 12935_2020_1695_MOESM3_ESM.doc]

Fig3F

| **SNU-387** | G0/G1 | S | G2/M |
| --- | --- | --- | --- |
| Si-NC | 54.95% | 33.42% | 11.63% |
| Si-circ_0061395 | 72.39% | 17.87% | 9.73% |

| **Huh7** | G0/G1 | S | G2/M |
| --- | --- | --- | --- |
| Si-NC | 52.04% | 35.59% | 12.37% |
| Si-circ_0061395 | 67.99% | 24.46% | 10.55% |

Fig5D

| **SNU-387** | G0/G1 | S | G2/M |
| --- | --- | --- | --- |
| Si-NC | 57.01% | 30.38% | 12.61% |
| Si-circ_0061395 | 74.64% | 12.91% | 12.45% |
| Si-circ_0061395+inhibitor NC | 73.24% | 16.64% | 10.12% |
| Si-circ_0061395+miR-877-5p inhibitor | 65.81% | 23.46% | 10.73% |

| **Huh7** | G0/G1 | S | G2/M |
| --- | --- | --- | --- |
| Si-NC | 49.16% | 38.23% | 12.61% |
| Si-circ_0061395 | 72.68% | 16.97% | 10.35% |
| Si-circ_0061395+inhibitor NC | 74.58% | 16.81% | 8.61% |
| Si-circ_0061395+miR-877-5p inhibitor | 60.21% | 28.97% | 10.81% |

Fig6M

| **Huh7** | G0/G1 | S | G2/M |
| --- | --- | --- | --- |
| miRNA NC | 54.05% | 32.30% | 13.65% |
| miR-877-5p mimic | 74.59% | 17.25% | 8.16% |
| miR-877-5p+pc-NC | 70.19% | 15.63% | 14.18% |
| miR-877-5p+pc-PIK3R3 | 62.04% | 26.96% | 11.00% |

| **Huh7** | G0/G1 | S | G2/M |
| --- | --- | --- | --- |
| miRNA NC | 50.29% | 35.88% | 13.82% |
| miR-877-5p mimic | 72.15% | 20.63% | 7.21% |
| miR-877-5p+pc-NC | 74.23% | 18.68% | 7.09% |
| miR-877-5p+pc-PIK3R3 | 56.91% | 33.18% | 9.91% |
